# Mixed hydraulic responses to drought in six common woody species from a dry evergreen sclerophyll forest in South Africa

**DOI:** 10.1093/treephys/tpaf045

**Published:** 2025-04-16

**Authors:** Robert P Skelton, Daniel Buttner, Alastair J Potts

**Affiliations:** School of Animal, Plant and Environmental Sciences, University of the Witwatersrand and South African Environmental Observation Network (SAEON), 1 Jan Smuts Ave., Johannesburg 2001, South Africa; Botany Department, Nelson Mandela University, Gqeberha 6031, South Africa; Botany Department, Nelson Mandela University, Gqeberha 6031, South Africa

**Keywords:** Albany Subtropical Thicket, drought responses, embolism, evergreen, hydraulic safety margin, plant functional traits, stomata, xylem water potential, xylem

## Abstract

Despite the emergence of a general conceptual framework for woody tree response to drought, few studies link variation in functional traits of coexisting species to drought outcomes in diverse plant communities. We use a natural drought event to test an ecological prediction from the embolism avoidance hypothesis: that co-existing species of a single growth form (woody trees) will converge upon traits that avoid embolism during all but the most severe droughts. We evaluated hydraulic traits and drought responses of six common woody tree species from South Africa’s Albany Subtropical Thicket. For each species, we measured laboratory-based xylem vulnerability and Pressure–Volume curves, and *in situ* minimum water potentials and four metrics of drought canopy damage during a dry period as well as a subsequent wetter period. We also quantified leaf construction and plant architecture traits, including tree height, Huber value and leaf mass per area (LMA). Species varied in the water potential associated with 50% loss of xylem function (P_50_), and turgor loss point, leading to between-species variation in stomatal and hydraulic safety margins. All species were shown to withstand leaf xylem water potentials more negative than −4.5 MPa before experiencing embolism. Predicted percent embolism during the dry period was associated with whole-plant drought damage but only following recovery. The LMA, modulus of elasticity, Huber value and tree height were also associated with drought damage, albeit less predictably so. Our results provide support for the embolism avoidance hypothesis and demonstrate how knowledge of species’ hydraulic traits can predict canopy dieback during drought events. However, our study also reveals mixed functional responses to drought within a single major growth form (i.e., woody trees) within a community that is composed of multiple growth forms, highlighting the complexity of predicting drought outcomes in diverse communities.

## Graphical Abstract

**Graphical Abstract:**
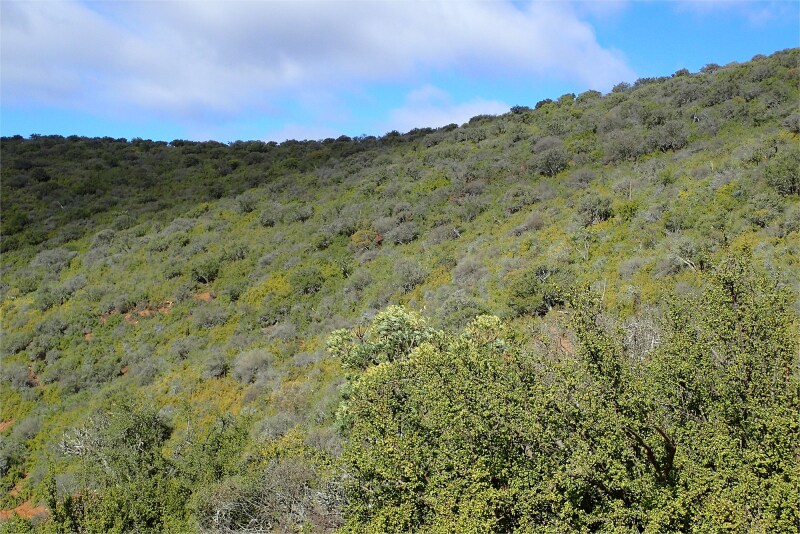

## Introduction

Increased rates of drought-induced tree mortality at global scales (Allen et al. 2010, Brodribb et al. 2020, Hammond et al. 2022) and associated alteration of community composition and function (McDowell et al. 2020) underscore the importance of understanding dynamic plant vascular function in response to dehydration. Such knowledge of plant vascular function can be used to improve predictions of the composition, productivity and resilience of plant communities under future climate conditions (Anderegg et al. 2015, Choat et al. 2018, Brodribb et al. 2020). Of importance in this regard is determining the capacity of the xylem to withstand hydraulic failure as plants desiccate, since maintaining hydraulic transport is associated with productivity and embolism is associated with loss of function and tissue dieback (Skelton et al. 2017b, Choat et al. 2018, Brodribb et al. 2020, 2021, McDowell et al. 2022).

Xylem physical tolerance to drought can be quantified from the relationship between xylem water potential and loss of xylem function (xylem vulnerability curves). Drought tolerance amongst species is typically compared using the xylem water potential value at which 50% loss of hydraulic conductance occurs (P_50_, MPa). Other xylem water potential reference points may also be used in specific physiological contexts; for example, the xylem water potential associated with the onset of embolism (P_12_), or severe hydraulic impairment (P_88_). P_100_ would equal the xylem water potential associated with complete xylem hydraulic failure, leading to desiccation and eventual mortality of the down-stream tissues. Such findings have been shown in herbaceous species (Brodribb et al. 2021) and conifers (Johnson et al. 2022). Inter-specific variation in P_50_ among conifer and angiosperm species has been shown to limit the dry end of species distributions along aridity gradients (Larter et al. 2017, Skelton et al. 2021). For example, among closely related woody conifer (Larter et al. 2017) and western north American oak species (Skelton et al. 2021), those occupying drier sites typically have less vulnerable xylem.

The risk of loss of hydraulic function can be assessed by coupling knowledge of plant xylem vulnerability to embolism with the most negative seasonal pressure potential (P_min_, measured in MPa). The water potential difference between P_min_ and P_50_ is referred to as the hydraulic safety margin (HSM, MPa), providing a useful proxy for the level of safety from extensive damage, and/or the extent of embolism within individuals (Delzon and Cochard 2014, Wagner et al. 2022). Since xylem embolism is associated with tissue damage (Skelton et al. 2017*a*, Brodribb et al. 2021) and plant mortality (Brodribb and Cochard 2009, Urli et al. 2013, Choat et al. 2018, McDowell et al. 2022), the distribution of HSM values might relate to the maintenance of productivity within communities.

Globally, Choat et al. (2012) found evidence of convergence in HSM for species across several of the world’s major biomes. Crucially, data availability of Pmin, P_50_ and P_88_ at broad spatial scales is scarce, both across and within species. A lack of high-quality hydraulic data limits our ability to understand patterns of productivity and mortality risk. This is especially true for African ecosystems, which contribute < 5% of all trait data to the global trait database (Choat et al. 2012), despite containing > 15% of global species diversity. An important gap that has been identified is a lack of high-quality hydraulic data. This gap includes the Albany Subtropical Thicket biome of South Africa, which occurs in a region of non-seasonal rainfall and common droughts spanning months to years (Vlok et al. 2003). Xylem trait data, such as P_50_ and HSM, can assist in our understanding of the ecology of this unique and understudied vegetation.

**Figure 1:**
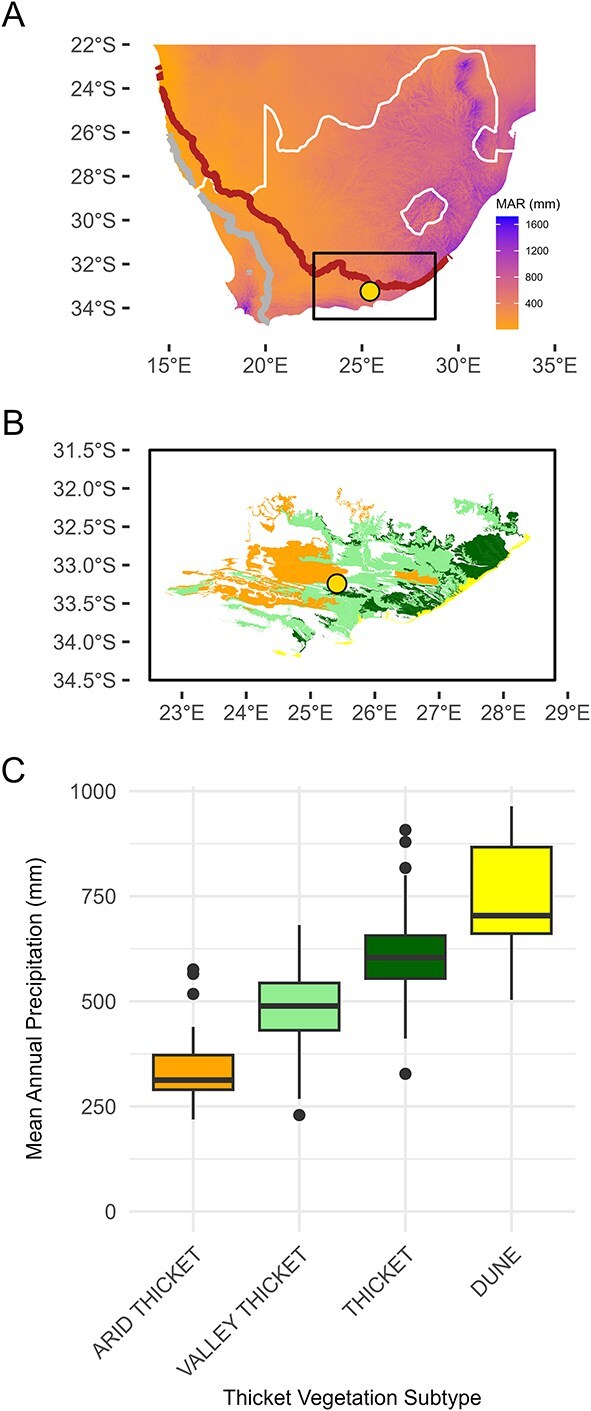
(A) Mean annual precipitation (MAP, mm) of southern Africa falls within two rainfall regimes, with a winter rainfall region (west of the grey line) and a summer rainfall region (east of the red line). The study site (circle) falls within the non-seasonal rainfall zone. (B) The Albany Subtropical Thicket biome, which is composed of various vegetation types, occurs predominantly in the non-seasonal rainfall zone. The location of the study site is also shown (circle). (C) Boxplot showing MAP in the Subtropical Thicket biome. Boxes indicate interquartile range, thick horizontal black line indicates median, and whiskers indicate full range of values.

The Albany Subtropical Thicket biome can be considered a dwarf forest rich in succulents—it has low stature (<3–5 m in height) and a closed canopy (Vlok et al. 2003). It is found only in South Africa, within a climatic region where droughts are a common environmental feature (Figure 1) (Mahlalela et al. 2020, Archer et al. 2022). Many of the canopy-dominant tree lineages in Thicket are associated with more mesic subtropical (or tropical) environments (Cowling 1983, Cowling et al. 2005) and drought is likely a strong filter shaping species occurrence in the various forms of thicket (Everard 1987). An important, and unexplained, feature of this vegetation is that it is dominated by evergreen species, in terms of diversity and phytomass, in a semi-arid region. In general, mesic thicket occurs around 586 ± 129 mm of mean annual precipitation (± SD), valley thicket 456 ± 100 mm and arid thicket 296 ± 94 mm (Figure 1) (Vlok et al. 2003). Precipitation is highly variable and unpredictable across the biome as it occurs within the meteorologically complex interface of the winter-rain bearing cold front systems in the west and summer-rain bearing low-pressure systems to the east (Engelbrecht et al. 2015), and intra- or inter-annual droughts can occur within any season or across a span of years (Archer et al. 2022). Drought is likely a key driver in this vegetation, but there has been very limited exploration into the drought ecology of the system and investigations have been largely restricted to the dominant and exceptionally drought-resistant succulent shrub *Portulacaria afra* (Guralnick and Ting 1986, 1987), or tree mortality across decades with drought as a purported driver (Lechmere-Oertel et al. 2005). Here we characterize species' drought responses and tolerances of six common tree species, and later discuss the ecological implications of these findings.

Our main objectives are (i) to test an ecological prediction from the embolism avoidance hypothesis, which posits that coexisting woody tree species will converge on functional trait combinations that help avoid embolism during all but the most severe droughts, (ii) to assess drought responses in Albany Subtropical Thicket vegetation, thereby contextualizing its drought tolerance and understanding drought’s role as an ecological driver in this system, and (iii) to determine if there are clear links between functional trait combinations and in-field drought responses. We posed three primary questions: (i) How much variation exists in drought resistance traits among woody species in this environment? (ii) Do plants in this dry environment show convergence in embolism-avoidance traits? (iii) How strongly linked are various functional traits with in-field drought responses? We hypothesized that woody tree species would exhibit similar drought tolerances, with limited variability in functional traits, leading to uniform drought responses within the community. Our study reveals mixed hydraulic responses to drought among six common, co-occurring woody evergreen species in the Albany Subtropical Thicket. Notably, it demonstrates that canopy dieback during severe drought events are shaped by complex and varying combinations of functional traits. Our findings not only support the embolism avoidance hypothesis but also advance our understanding of how drought outcomes in diverse plant communities can be anticipated by examining specific functional trait combinations.

## Materials and methods

### Study site and sample species

Our study site was located at Kaboega Game Farm (33°15′40″S 25°20′16″E), situated in the Zuurberg mountain range of the Eastern Cape province (Figure 1). The reserve has a mean annual precipitation of 338 mm with a 35% coefficient of variation (based on a rainfall record of 64 years spanning 1948 to 2011). The minimum and maximum annual rainfall amounts across the available record are 134 and 668 mm, respectively. The property is topographically and geologically variable with Witteberg shales dominating the valley slopes and bottomlands and quartzite-derived sandstone on valley crests. The property has a range of vegetation types, including Fynbos, Nama-Karoo, Forest and Albany Subtropical Thicket; the boundaries amongst the biomes are largely dictated by geology, aspect, fire and frost (Cowling and Potts 2015, Duker et al. 2015). Thicket vegetation is largely restricted to the shales on the slopes*.* Here we only focused on trees growing in two thicket types: Baviaans Valley Thicket and Sunday's Arid Thicket (Dayaram et al. 2019).

We assessed six study species. *Boscia oleoides* (Burch. ex DC.) Toelken is a woody tree or large shrub up to 4 m and has the most restricted distributional range of all the species (Figure S1 available as Supplementary data at *Tree Physiology* Online). *Searsia longispina* (Eckl. & Zeyh.) Moffett is a small tree or rounded shrub endemic to South Africa. Its range spans the west coast, through the Great Karoo and into the southern coastal lowlands of the Eastern Cape. *Euclea undulata* Thunb. is a woody shrub or small tree widespread across southern Africa (including Namibia, Botswana, Swaziland and Zimbabwe) and most of South Africa (absent only from the central highveld and the West Coast). Outside of Thicket it is usually found on rocky slopes, or along watercourses in drier areas. *Schotia latifolia* Jacq. is a small to medium-sized tree that occurs in the coastal lowlands up to the base of the Great Escarpment across the Western Cape, Eastern Cape and KwaZulu-Natal provinces. *Pappea capensis* Eckl. & Zeyh. is a small- to medium-sized tree that occurs throughout the subtropical zones of Africa and onto the Arabian Peninsula. However, there is a southern genetic lineage that occurs along the coastal lowlands from the Western Cape province through KwaZulu-Natal province (Potts 2012). *Polygala myrtifolia* L. is an evergreen shrub or small tree found along the southern and south-eastern coasts. Although all these species are common in the Albany Subtropical Thicket, their distributions do differ amongst the Thicket subtypes, where *Boscia* and *Searsia* are largely absent from the mesic thicket, and *Schotia* and *Polygala* are largely absent from the arid thicket (Table 1).

**Table 1:** The mean annual precipitation and climatic water deficit of six common tree species found in the Albany Subtropical Thicket and their associations with the three thicket subtypes and presence in other biomes. The mean, and 5th and 95th percentile values (in brackets) and sample size (*n*) are given for MAP and CWD. See the supplementary information for further information.

		MAP (mm)		CWD (mm)		Thicket subtype			
**Species**	**Family**	**SA**	**Thicket**	**SA**	**Thicket**	**Arid**	**Valley**	**Mesic**	**Other biome associations**
*Boscia oleoides*	Capparaceae	420 (284–616) *n* = 43	475 (297–627) *n* = 28	957 (562–1136) *n* = 43	891 (559–1135) *n* = 28	Y	Y	N	Nama-Karoo
*Searsia longispina*	Anacardiaceae	492 (366–627) *n* = 63	506 (377–627) *n* = 41	804 (512–1109) *n* = 63	759 (506–943) *n* = 41	Y	Y	N	Nama-Karoo; Succulent Karoo
*Euclea undulata*	Ebenaceae	513 (300–663) *n* = 381	542 (348–663) *n* = 201	789 (473–1127) *n* = 381	709 (443–1092) *n* = 201	Y	Y	Y	Fynbos; Nama-Karoo; Succulent Karoo; Grassland; Savanna
*Pappea capensis*	Sapindaceae	549 (293–956) *n* = 226	480 (299–661) *n* = 134	777 (401–1139) *n* = 226	805 (468–1154) *n* = 134	Y	Y	Y	Savanna; Forest
*Schotia latifolia*	Fabaceae	619 (494–853) *n* = 89	608 (496–854) *n* = 54	588 (233–827) *n* = 89	614 (235–833) *n* = 54	N	Y	Y	Forest
*Polygala myrtifolia*	Polygalaceae	822 (353–1409) *n* = 1363	592 (308–852) *n* = 308	502 (209–1075) *n* = 1363	615 (381–1115) *n* = 308	N	Y	Y	Fynbos; Forest

### Study site conditions

**Figure 2:**
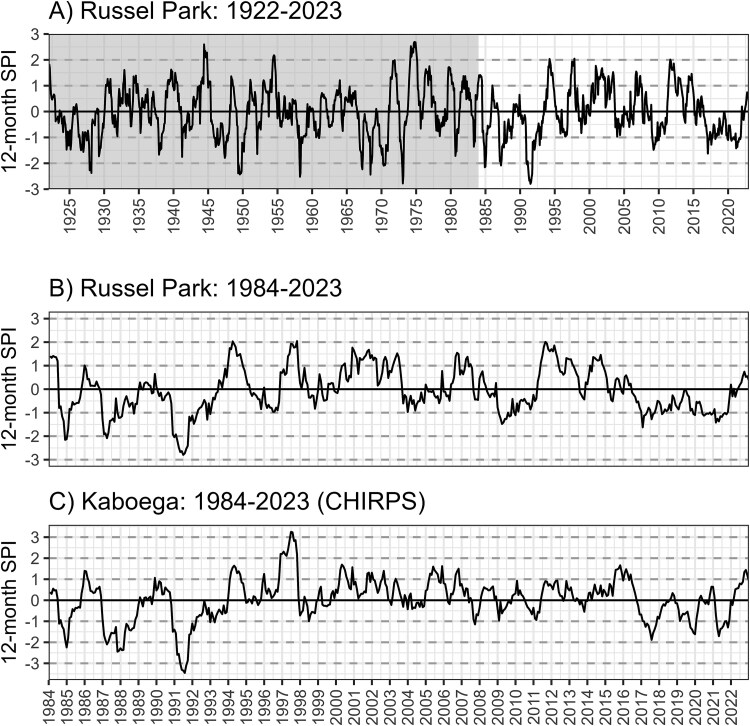
Long-term 12-month SPI values, based on measured rainfall data at Russel Park (A, B), and modelled rainfall data at the Kaboega study site (C).

The standard precipitation index (SPI) was used to characterize the drought and post-drought period (Figure 2). Monthly precipitation data were used from two sources: (i) a long-term rainfall dataset collected at the Russel Park Farm main house (spanning 1922 to 2023), which is situated ~ 25 km NEE of the study site, and (ii) the interpolated high-resolution CHIRPS rainfall dataset (available from the Climate Hazards Group and spanning 1984 until present; Funk et al. 2014). The SPI was calculated using the SPEI v1.8.1 library (Beguería and Vicente-Serrano 2023) in R 4.2.3 (R Core Team 2023).

### Estimation of leaf dry mass per area and Huber value

Leaf mass per area (LMA, g m^−2^) was determined for three individuals of each of the six focal species in August 2023, using two replicates of 10 leaves per plant and three plants per species. For each individual, 10 leaves were removed from twigs and imaged on a flatbed scanner at 300 d.p.i. Leaf projected area was determined from the scanned images with ImageJ 1.48v software (NIH, Bethesda, MD, USA, https://imagej.nih.gov/ij/). Leaves were thereafter oven-dried at 70 °C for 48 h, and their dry mass was determined. From these measurements, LMA was calculated (Poorter et al. 2009).

To calculate Huber value (sapwood area/leaf area) we sampled terminal, sun-exposed shoots from the most recent year’s growth from the outer canopy. This approach maximizes the likelihood that all the sapwood in the branch is still functional. Care was taken to select shoots that had not lost leaves or parts of leaves to mechanical damage, herbivory or early senescence and abscission. The sapwood area was calculated from the diameter of the sapwood following removal of the bark, while the total leaf area subtended by the stem area was quantified using the same method as outlined above. Individual leaf area was calculated as the total leaf area of all leaves divided by the total number of leaves.

### Leaf xylem water potential

Leaf xylem water potential (Ψ_md_, MPa) was measured at midday between 12:00 and 14:00 h on two separate periods. The first sampling period was from late September (first sample date: 29 September 2020) to early October (last sample date: 10 October 2020) during the protracted dry period. The second sampling period was in November 2022 during a wetter phase following the dry phase. At each time point, Ψ_md_ was measured on at least six representative individuals of each species. Samples of apical shoots from fully sun-exposed branches were wrapped in moist paper towel, clipped and immediately sealed in a humidified zip-lock bag. Water potential readings were taken without delay (<5 min after being severed from the tree) with a Scholander-type Pressure Chamber (Model 600; PMS Instruments Company, Corvallis, OR, USA).

### Leaf xylem vulnerability curves

Leaf xylem vulnerability to embolism was measured using the optical vulnerability technique (Brodribb et al. 2016). Branches were selected from three healthy and well-hydrated individuals for each of the six focal species and were cut in the early hours (c. 06:00 to 07:00 h) to ensure greater hydration at the time of sampling. All selected branches exceeded 1 m in length to avoid creating open vessels. The excised end of the branch was recut under water at 10 cm from the cut end to further reduce xylem tension. Branches were then immediately transferred to large, humidified plastic bags to reduce evapotranspiration and maintain hydration of the sample during transport back to the laboratory at Nelson Mandela University.

Samples were allowed to dehydrate slowly on a workbench in a darkened room for a period of a few days while we observed embolism formation in a healthy, fully expanded distal leaf. To do so, we captured sequential images of the leaf xylem network using custom-built 3D-printed optical leaf clamps equipped with a ×20 magnifier and 8-mega-pixel digital camera (Camera Module v2, Raspberry Pi Foundation, UK) and an LED light to illuminate the sample (for an overview and details of methods and materials used see http://www.opensourceov.org). The camera was programmed to capture images at 5 min Intervals, until embolism events were no longer detected (c. 48–168 h, dependent on species).

Simultaneously, stem xylem water potential was monitored using a psychrometer (ICT International, Armidale, NSW, Australia) installed within 20 cm of the leaf. Xylem water potential was automatically logged at 10 minute intervals. To evaluate whether recorded stem xylem water potential provided an accurate assessment of leaf xylem water potential values we periodically also took leaf xylem water potential measurements using a Scholander-type Pressure Chamber (Model 600; PMS Instruments Company, Corvallis, OR, USA). Stem and leaf xylem water potentials were consistently found to be within 0.1 MPa of each other, indicating that branches were in equilibrium.

Embolism identification and quantification was carried out using the methods outlined in Brodribb et al. (2016). Briefly, the image stack was loaded into ImageJ, and the difference in pixels between successive images was recorded. We then manually filtered the pixel difference stack and removed pixels resulting from obvious leaf movement. Leaf embolism was measured as the cumulative sum of embolized pixels of images and then converted to a percentage of the total embolized pixels of the complete dried sample. A full account of the procedures, software and technique are available at http://www.opensourceov.org.

The percentage of leaf xylem embolized was plotted against water potential and fitted using a sigmoidal function:

Percent embolism = 100 − 100/(1 + e^a(Ψ −b)^),

where *a* corresponds to the sensitivity to decreasing water potential (proportional to the slope of the equation) and *b* is the water potential associated with 50% embolism. We also extracted the water potentials associated with 12% and 88% embolism.

### Pressure–Volume Curves and TLP

To determine a proxy for the point of stomatal closure, we measured the water potential corresponding to bulk leaf turgor loss (TLP; MPa). Previous studies have shown that stomatal aperture is reduced significantly at leaf TLP (Brodribb and Holbrook 2003, Buckley et al. 2003). The point of bulk leaf cell turgor was determined for individuals of each study species by the relationship between Ψ_leaf_ and water content in the leaf. Branches from well-hydrated individuals of each study species were collected between March and May 2021 cut underwater and allowed to hydrate to water potentials less negative than −0.1 MPa. From these branches, at least three leaves per species were removed and used to quantify leaf Pressure–Volume curves using the bench-drying technique (Tyree and Hammel 1972). The Ψ_leaf_ values and leaf weight were measured periodically until Ψ_leaf_ stopped declining or desiccation-induced cell damage was observed in the leaves. At this stage, leaves were placed in a drying oven at 70 °C for at least 48 h for complete desiccation to determine dry weight. For each leaf, relative water content was determined and plotted against Ψ_leaf_, and the Ψ_leaf_ at turgor loss was determined as the point of inflection between the linear and nonlinear portions of the plot. A mean TLP ± standard error (*n* = 3) was calculated for each species. The bulk elastic modulus (ε) and osmotic potential at full turgor (Ψ_osm_**,** MPa) were also quantified from the Pressure–Volume curves. The bulk elastic modulus of an individual leaf represents the ratio of the change in cell turgor (P) to that in the relative cell volume (ΔV/V) of the leaf:

ε = ΔP/(ΔV/V).

### Predicted percent embolism, and hydraulic and stomatal safety margins

The predicted amount of embolism that each individual would have experienced in the field was calculated using the mean vulnerability curve for the species together with the minimum water potential recorded for that individual. Hydraulic safety margin (HSM, MPa) was calculated as the difference between the water potential associated with 50% observed embolism in the leaf (leaf P50) and the minimum leaf xylem water potential observed in either a dry period (September 2020, HSM 2020) or in a wetter period (November 2022, HSM 2022). Stomatal safety margin (SSM, MPa) was calculated as the water potential difference between leaf P50 and the turgor loss point. We also calculated hydraulic and stomatal safety margins from the water potentials associated with 12% and 88% observed embolism in the leaf.

### Drought damage surveys

We assessed individual plant condition through four damage metrics designed to scale from the leaf to the canopy (whole individual plant) level (Stone et al. 2008, Losso et al. 2022). Leaf discoloration was defined as the colour of each leaf from brown (dead) through orange and yellow to green. The “dead leaves” metric was defined as the percentage of dead leaves across the entire canopy. The “foliage” metric was defined as the percentage of each branch covered in leaves averaged across the entire canopy. The “crown extent” metric was defined as the proportion of canopy still alive relative to the potential complete canopy (i.e., in the absence of abiotic stress and leaf dieback). For each of the ten trees per species, two observers visually assessed the tree for each of the four metrics, generating an average score for each metric. Assessments were conducted in early October of 2020 and again, on the same trees, in late August 2022.

### Statistical analysis

To test for differences in LMA, Huber value, TLP, ε, Ψ_osm_, P_12_, P_50_, and slope of the vulnerability curve between species, ANOVAs, in addition to a post hoc Tukey’s honestly significant difference tests, were used. Linear models were used to determine the relationship between single trait predictors (e.g., HSM, predicted percent embolism) and damage metrics. In addition, generalized linear models (GLMs) were employed to predict each drought outcome based on combinations of eight predictor variables: P_50_, TLP, ε, Ψ_osm_, leaf mass area (LMA), plant height, Huber value, and predicted percent embolism (PPE), using the Gaussian family with a log link function. As there were more descriptors (i.e., traits) than objects (species), it was not possible to run a GLM with all the traits included simultaneously. Thus, multiple GLMs were constructed using different combinations of fewer traits, specifically all combinations of traits up to three predictors were identified, and a GLM was run for each combination using the ‘glm’ function in the lme4 package.

To evaluate the GLMs, Akaike Information Criterion (AIC) scores were calculated for each GLM, and models were ranked in ascending order of AIC values. To determine whether differences in AIC were meaningful, the ΔAIC (delta AIC) was computed by subtracting the lowest AIC value from each model’s AIC. Models with ΔAIC ≤ 2 were considered to have similar levels of support. Akaike weights were then calculated to estimate the relative likelihood of each model, allowing for a quantitative comparison of model plausibility. The best-fit model was selected as the model with the lowest AIC and highest Akaike weight, provided that no collinearity existed among its predictor variables. Collinearity was assessed using variance inflation factors (VIFs) calculated with the ‘vif’ function in the car package, and models with VIF values exceeding 10 were excluded. This threshold was chosen because the predictor variables are plant functional traits that are expected to naturally co-vary due to shared physiological and ecological roles.All statistical analyses were conducted in R 4.2.3 (R Core Team 2023).

## Results

### Leaf dry mass per area and Huber value

Leaf dry mass per area (LMA, g m^−2^) varied threefold between our six sample species (Table 2), with *Searsia* having the lowest LMA and *Boscia* having the highest values of all six species. *Pappea* and *Schotia* had greater total leaf area per shoot than *Searsia*, *Euclea* and *Polygala* (Table 2). *Pappea* had a higher Huber value compared with other sample species (Table 2). Mean individual leaf area was greater in *Schotia* compared with *Polygala*, but similar across most of the remaining species (Table 2).

**Table 2:** Species characteristics, including maximum height under optimal environmental conditions (max. Height), and traits measured from plants in the study area: height, canopy width, number of stems, total stem area (emergent stems at 15 cm), leaf mass area, mean leaf area, total leaf area per shoot (~30 cm in length) and Huber value. Values are mean ± SE. with sample sizes (n; individual plants) provided in parentheses. Species are ordered from highest to lowest mean annual precipitation associated with the distribution within South Africa. Letters indicate statistical differences (*P* < 0.05).

		At study site							
	Max. height (m)	Height (m) (*n* = 10)	Canopy width (m) (*n* = 10)	No of stems (min–max) (*n* = 10)	Total stem area (cm^2^) (*n* = 10)	LMA (g m^−2^) (*n* = 3)	Mean leaf area (cm^2^)	Total leaf area per shoot	Huber value
*Boscia oleoides*	10	3.6 ± 0.1	2.8 ± 0.2	1	363 ± 64	346.1 ± 56.8 (3) a	NA	NA	NA
*Searsia longispina*	5	2.2 ± 0.2	3.3 ± 0.2	>10	†	106.1 ± 7.6 (3) d	0.59 ± 0.1 (2) ab	92.3 ± 3.7 (2) a	0.144 ± 0.001 (2) a
*Euclea undulata*	7	2.4 ± 0.2	2.7 ± 0.3	2–9	288 ± 100	265.9 ± 15.7 (3) ab	0.54 ± 0.27 (5) ab	128.0 ± 6.9 (5) a	0.136 ± 0.03 (5) a
*Pappea capensis*	8	3.7 ± 0.1	4.2 ± 0.3	1–5	409 ± 75	136.5 ± 10.9 (3) cd	0.60 ± 0.07 (5) ab	185.0 ± 10.8 (6) b	0.379 ± 0.06 (6) b
*Schotia latifolia*	15	2.6 ± 0.3	3.1 ± 0.5	1–11	222 ± 75	218.0 ± 13.5 (3) bc	0.83 ± 0.14 (5) b	183.0 ± 9.5 (5) b	0.114 ± 0.03 (5) a
*Polygala myrtifolia*	4	1.4 ± 0.1	0.9 ± 0.2	1–6	9 ± 2	144.0 ± 9.7 (3) cd	0.22 ± 0.06 (5) a	131.0 ± 9.2 (5) a	0.127 ± 0.03 (5) a

†unable to access the stems.

### Xylem vulnerability to embolism

**Figure 3:**
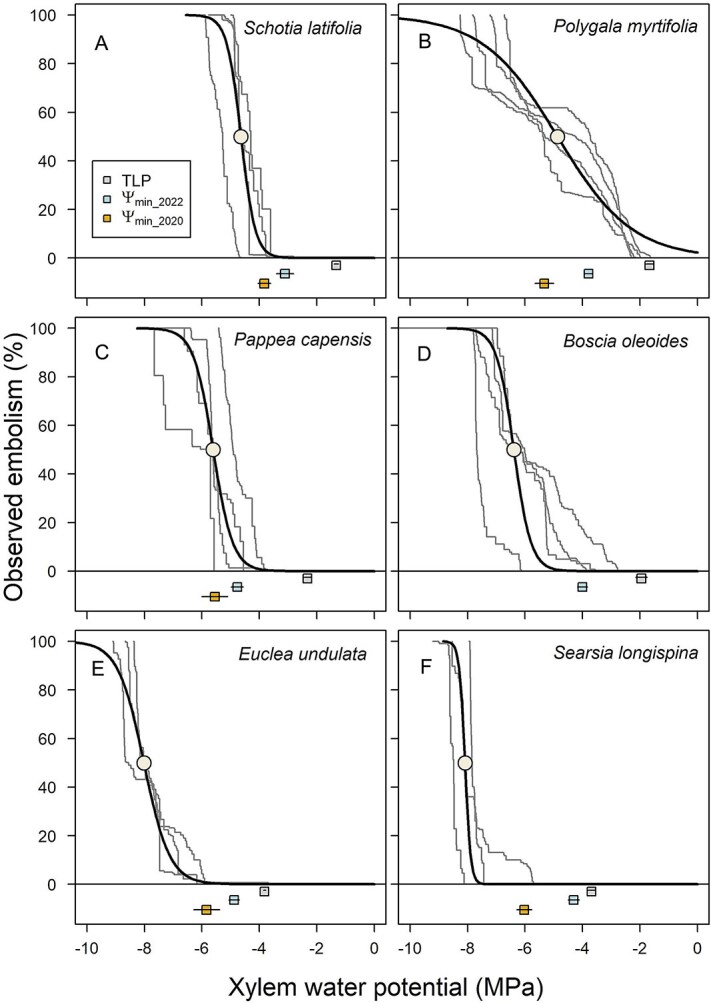
Leaf xylem vulnerability to embolism for six dominant woody tree species from Albany Subtropical Thicket. Thin grey lines indicate traces for individuals and thicker black lines indicate the species mean. P_50_ values for each species are shown as circles. Also shown is the turgor loss point (TLP, mean ± SE, MPa), and mean midday leaf xylem water potential measured during the dry august 2020 and the wetter November 2022 (Ψ_min_2020_ and Ψ_min_2022_, mean ± SE, MPa).

**Figure 4:**
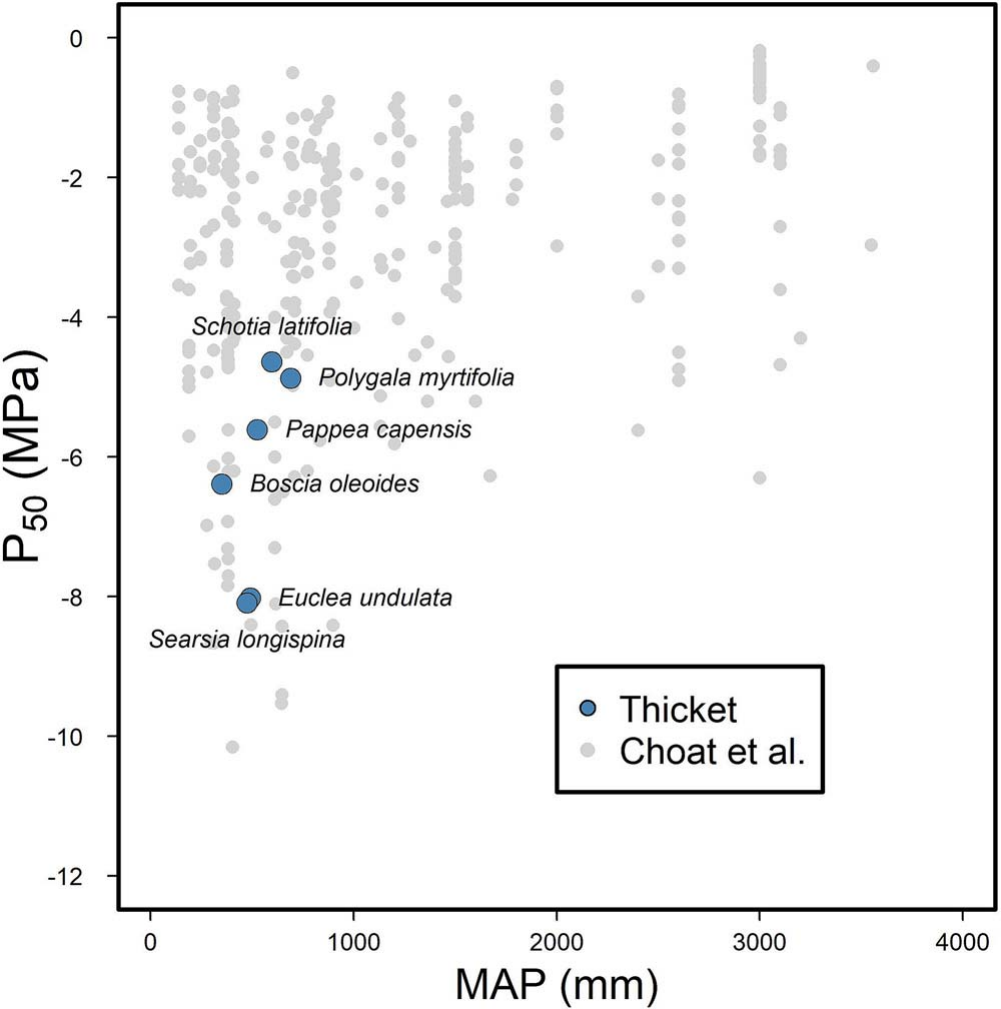
Relationship between xylem capacity to withstand air entry (mean P_50_ ± SE, MPa) and mean annual precipitation (MAP, mm) associated with species’ distribution for six study species (large circles) and angiosperm species from the global xylem functional traits database (grey circles).

We observed significant variation in xylem vulnerability curve parameters between sample species (Figure 3), including almost twofold variation in leaf xylem P_50_ between the most vulnerable (*Schotia* = −4.64 ± 0.24 MPa) and the least vulnerable species (*Searsia* = −8.09 ± 0.21 MPa) (F_5_ = 24.6; *p* = 5.5 × 10^−7^; Table 3). All species were able to withstand leaf xylem water potentials more negative than −4.5 MPa before experiencing > 50% embolism. *Schotia* and *Polygala* (P_50_ = −4.87 ± 0.22 MPa) had the most vulnerable leaf xylem, while *Searsia* and *Euclea* (P_50_ = −8.02 ± 0.13 MPa) had the most resistant xylem (Figure 3; Table 3). The two species in our dataset with the greatest capacity to withstand embolism —*Searsia* and *Euclea—*possess leaf P_50_ values that are only surpassed by species from 14 other angiosperm genera (out of 526) in the global xylem functional traits database (Choat et al. 2012; Figure 4). All those more resistant genera/species from the global database occur in semi-arid or Mediterranean-type ecosystems with dry summers (Choat et al. 2012).

**Table 3:** Physiological traits extracted from Pressure–Volume and xylem vulnerability to embolism curves for each species. All reported values are mean ± standard error, with sample size in parentheses. Significant differences within each trait are shown using different lowercase letters.

**Species**	**TLP (MPa)**	**Ψ**_**osm**_ **(MPa)**	**ε (MPa)**	**Leaf P_50_ (MPa)**	**Slope**
*Boscia oleoides* (Capparaceae)	−1.95 ± 0.20 (3) bc	1.37 ± 0.11	4.61 ± 0.24	−6.39 ± 0.41 (4) b	−3.34 ± 1.85 a
*Searsia longispina* (Anacardiaceae)	−3.69 ± 0.11 (3) d	2.17 ± 0.22	6.14 ± 0.94	−8.09 ± 0.21 (3) c	−10.07 ± 3.38 b
*Euclea undulata* *(*Ebenaceae)	−3.82 ± 0.04 (3) d	2.92 ± 0.13	12.43 ± 1.58	−8.02 ± 0.13 (3) c	−2.23 ± 0.06 a
*Pappea capensis* (Sapindaceae)	−2.33 ± 0.11 (4) c	1.45 ± 0.11	3.88 ± 0.88	−5.61 ± 0.34 (4) ab	−2.93 ± 0.47 a
*Schotia latifolia* (Fabaceae)	−1.33 ± 0.08 (3) a	0.93 ± 0.09	3.87 ± 0.38	−4.64 ± 0.24 (4) a	−4.03 ± 0.15 ab
*Polygala myrtifolia* *(*Polygalaceae)	−1.67 ± 0.12 (3) ab	1.02 ± 0.05	3.48 ± 0.85	−4.87 ± 0.22 (4) a	−0.77 ± 0.08 a

### Turgor loss point

We observed some variation in the water potential at turgor loss (TLP, MPa) among species (F_5_ = 72.7; *p* = 4.9 × 10^−9^; Table 3). *Schotia* (−1.33 ± 0.08 MPa), *Boscia* (−1.95 ± 0.2 MPa) and *Polygala* (−1.67 ± 0.12 MPa) all lost turgor at water potentials less negative than −2 MPa, while *Searsia* (−3.69 ± 0.11 MPa) and *Euclea* (−3.82 ± 0.04 MPa) both lost turgor at water potentials more negative than −3 MPa. There was a strong, negative linear relationship between log transformed modulus of elasticity and TLP (r^2^ = 0.73, *p* = 0.03), with the two species with the most negative TLP, *Searsia* and *Euclea*, exhibiting higher modulus of elasticity than the remaining four species, which all had similar modulus of elasticity values (Table 3). We observed no relationship between LMA and either xylem vulnerability to embolism or TLP (Figure S2 available as Supplementary data at *Tree Physiology* Online).

### Stomatal and hydraulic safety margins

When we combined the TLP with the leaf xylem vulnerability curves to define stomatal safety margin (SSM, MPa), our results showed that all species lost turgor prior to experiencing embolism (Table 4 and Table S1 available as Supplementary data at *Tree Physiology* Online; Figure 3). Further, two types of responses emerged in our group of six woody tree species: *Schotia*, *Pappea* and *Polygala* had similar, narrower SSMs (less than 4 MPa) versus *Boscia*, *Euclea*, and *Searsia,* which had similar, large SSMs (>4 MPa) (Table 4). *Schotia* and *Polygala* lost turgor at the least negative water potentials, and had the least resistant xylem, while *Pappea* lost turgor at intermediate water potentials, but also had intermediate xylem P_50_ (Table 4). *Boscia* also had intermediate xylem P_50_, but lost turgor at less negative water potentials, while *Euclea* and *Searsia* lost turgor at more negative water potentials and had very resistant xylem (Table 4).

**Table 4:** Hydraulic and stomatal safety margins for species. Values are mean ± SE, with sample size in parentheses. Different letters denote significant differences between species at *p* < 0.05; each trait (per year for HSM) was analysed statistically using one-way ANOVA and post-hoc Tukey HSD.

**Species**	**HSM 2020 (MPa)**	**HSM 2022 (MPa)**	**SSM (MPa)**
*Boscia oleoides* (Capparaceae)	NA	2.39 ± 0.17 (7) bc	4.44 ± 0.20 b
*Searsia longispina* (Anacardiaceae)	2.06 ± 0.26 (10) b	3.78 ± 0.19 (7) d	4.40 ± 0.11 b
*Euclea undulata* *(*Ebenaceae)	2.18 ± 0.45 (11) b	3.14 ± 0.18 (7) cd	4.20 ± 0.04 b
*Pappea capensis* (Sapindaceae)	0.06 ± 0.45 (10) a	0.84 ± 0.21 (7) a	3.28 ± 0.11 a
*Schotia latifolia* (Fabaceae)	0.82 ± 0.22 (10) ab	1.53 ± 0.30 (8) ab	3.31 ± 0.08 a
*Polygala myrtifolia* *(*Polygalaceae)	−0.46 ± 0.33 (10) a	1.08 ± 0.15 (7) a	3.20 ± 0.12 a

Our snapshot water potential measurements taken during a protracted dry period (in 2020) showed that *Polygala* and *Pappea* experienced water potentials that could have resulted in substantial embolism in the leaf xylem (59.0 ± 5.8% and 55.7 ± 14.0%, respectively). Accordingly, both species experienced water potentials resulting in a HSM from P_50_ < 0 MPa (Table 4). *Schotia* experienced water potentials that could have resulted in 12.7 ± 4.3% embolism in the leaf xylem (HSM from P_50_ > 0 MPa, HSM from P_12_ < 0 MPa; Table 4 and Table S1 available as Supplementary data at *Tree Physiology* Online). *Euclea* and *Searsia* had large HSMs and were unlikely to have experienced embolism in their leaf xylem (Table 4 and Table S1 available as Supplementary data at *Tree Physiology* Online). These observations contrasted with the snapshot xylem water potential readings taken during a wetter period (in 2022), in which all species were shown to have maintained positive HSM from P_50_ (Table 4), suggesting they were unlikely to have experienced > 50% embolism in the leaf xylem. However, during the wetter period *Polygala* and *Pappea* experienced water potentials that could have resulted in some embolism in the leaf xylem (30 ± 5.8% and 12.6 ± 14.0%, respectively) (Table S1 available as Supplementary data at *Tree Physiology* Online). Accordingly, both species had HSM from P_12_ < 0 MPa.

### Drought responses

**Figure 5:**
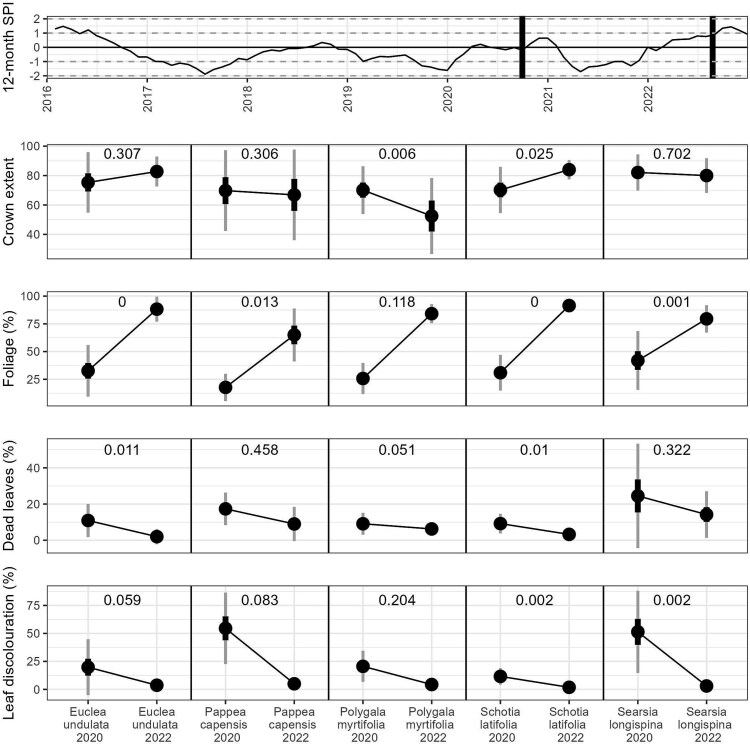
Assessment of drought damage and recovery for the six study species. The assessment of four metrics were taken at two periods—at the end of a long dry phase (October 2020), and after 6 months of above-average rainfall (August 2022). The mean percentage (points), standard error (black bars) and standard deviation (grey bars) are shown. *P*-values from a paired T-test are shown in each sub-plot.

Assessments of individual plant conditions were conducted during the protracted dry period (in early October 2020) and again, on the same trees, during a wetter period (in late August 2022, Figure 5). Leaf discolouration was higher in all species during the dry period compared with the wetter period, although only significantly higher in *Schotia* and *Searsia* (Figure 5). *Searsia* and *Pappea* had the highest leaf discolouration (both > 50%) during the dry years in comparison with all other species (Figure 5). All species had more dead leaves during the drier period, although this difference was only significant for *Schotia* and *Euclea*, and marginal (*p* = 0.051) for *Polygala* (Figure 5). The percentage of dead leaves was highest during the dry period in *Pappea* (~20%) and Searsia (>20%) and remained high in both species following rainfall (>10%) (Figure 5).

Four of the five species had significantly less foliage along their branches during the drier period compared to the amount of foliage present following rainfall, although the recovery trend was consistent in all five species (Figure 5). Despite the recovery in foliage following rainfall in *Pappea*, the percentage of foliage across the canopy remained relatively low (<70%) when compared with the remaining four species (Figure 5). The patterns observed in crown extent were more variable amongst species (Figure 5). Crown extent in *Pappea* and *Polygala* was low in comparison with other species during the dry period (~70%) and remained as low in *Pappea* and decreased further (to < 60%) in *Polygala* following rainfall (Figure 5). The opposite trend was observed in *Schotia* and *Euclea*, where crown extent was lower during the drought period compared with the wetter period, although only significantly so in *Schotia* (Figure 5). Crown extent in *Searsia* displayed no significant change between the drier and wetter periods, remaining high during both periods (>80%) (Figure 5).

### Relationships between predicted percent embolism and damage metrics

The HSM from P_50_ was not significantly related to any of the damage metrics during the drought, and was only marginally associated with crown extent (R^2^ = 0.75, *p* = 0.059) following recovery of the vegetation (Figure S3 available as Supplementary data at *Tree Physiology* Online). The strength of the relationships between drought damage improved if we considered HSM from P_12_, although it was only significantly related to the damage metrics following recovery (Figure S4 available as Supplementary data at *Tree Physiology* Online). The HSM from P_12_ was significantly positively associated with crown extent (R^2^ = 0.9, *p* = 0.013), and negatively associated with dead leaves (R^2^ = 0.78, *p* = 0.047) and leaf discolouration (R^2^ = 0.93, *p* = 0.009) following recovery of the vegetation. P_50_ was only marginally associated with crown extent (R^2^ = 0.76, *p* = 0.053) during the drought, and was not related to any of the damage metrics following recovery (Figure S5 available as Supplementary data at *Tree Physiology* Online).

**Figure 6:**
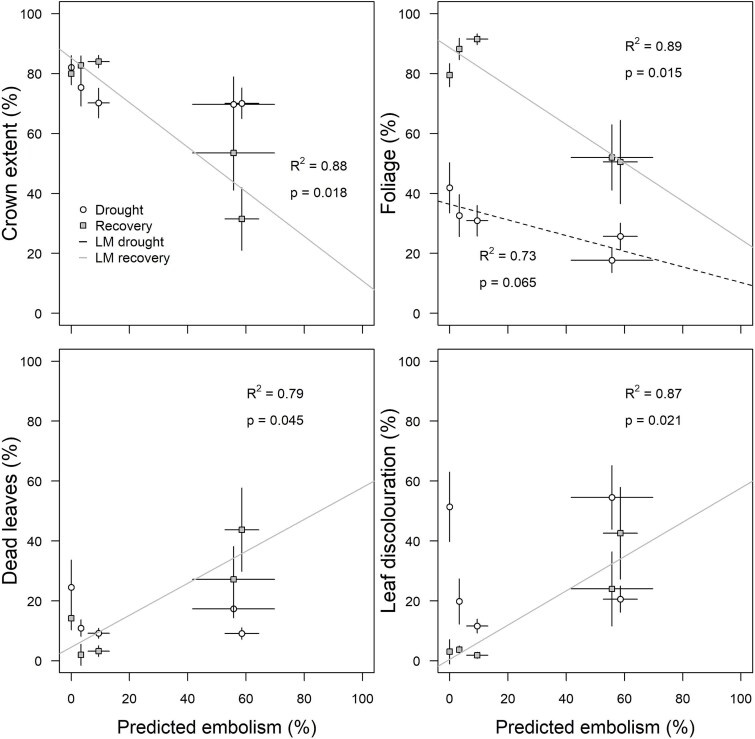
Assessment of the relationships between predicted percent embolism during the drought (mean ± SE) and four drought damage and recovery metrics (mean ± SE) for the five study species. Also shown are the significant (solid lines) and marginally significant (dashed lines) results of the linear models of the relationships between these variables for each observation period. The relationships between other physiological traits, such as HSM and P_50_, and the in-field metrics are available in the supplementary material, but did not show significant relationships.

The predicted percent embolism based on the minimum water potential during the drought period was not significantly associated with crown extent, dead leaves or leaf discolouration during the drought period, and was only marginally positively related to defoliation (R^2^ = 0.73, *p* = 0.065; Figure 6). However, predicted percent embolism was significantly associated to all four damage metrics during the recovery period (Figure 6). We observed negative relationships between predicted percent embolism during the drought and crown extent (R^2^ = 0.88, *p* = 0.018) and foliage (R^2^ = 0.89,   *p* = 0.015) following recovery of the vegetation. We also detected significant positive relationships between predicted percent embolism during the drought and dead leaves (R^2^ = 0.79, *p* = 0.045), and leaf discolouration (R^2^ = 0.87, *p* = 0.021).

When multiple traits were considered, functional trait combinations were shown to predict drought outcomes in both sampling periods (Table 5). In the drier period (October 2020), crown extent was negatively related to P_50_ (coefficient = −6.64, standard error = 0.49). Foliage showed a negative relationship with LMA (coef. = −0.05, SE = 0.002), height (coef. = −4.04, SE = 0.17) and predicted percent embolism (coef. = −0.3, SE = 0.01). The proportion of dead leaves exhibited a negative association with P_50_ (coef. = −6.08, SE = 0.02) and modulus of elasticity (coef. = −2.16, SE = 0.01), while showing a positive association with height (coef. = 2.00, SE = 0.03). Leaf discolouration was negatively associated with both modulus of elasticity (coef. = −10.98, SE = 0.22) and osmotic potential at full turgor (coef. = −50.81, SE = 0.94), and positively related to the Huber value (coef. = 63.89, SE = 2.65).

**Table 5:** Results for generalized linear models (GLM) to predict each drought outcome (crown extent, foliage, dead leaves, leaf discolouration) based on combinations of eight predictor variables: P50, TLP, modulus of elasticity (MOE), osmotic potential at full turgor (Osm. Pot.), leaf mass area (LMA), height, Huber value (HV) and predicted percent embolism (PPE). In each case results for the best fit formula are shown, with significant predictors shown in bold font. The number of observations (species) was five in all models.

**Season**	**Formula**	**AIC**	**McFadden R** ^**2**^	**Deviance explained**	**No. of predictors**
Drier	Crown extent ~P50^*^ + Osm. Pot.^ns^ + HV	9.98	0.99	0.99	3
	Foliage ~LMA^*^ + Height^*^ + PPE^*^	3.70	0.99	0.99	3
	Dead leaves ~P50^**^ + MOE^**^ + Height^**^	−13.93	0.99	0.99	3
	Discoloration ~MOE^*^ + Osm. Pot.^*^ + HV^*^	9.55	0.99	0.99	3
Wetter	Crown extent ~HV^*^ + Height^**^ + PPE^**^	−0.73	0.99	0.99	3
	Foliage ~P50^*^ + HV^**^ + Height^**^	1.41	0.99	0.99	3
	Dead leaves ~Osm. Pot.^**^ + HV^*^ + Height^*^	12.56	0.99	0.99	3
	Discoloration ~P50^*^ + Height^**^ + PPE^**^	−8.80	0.99	0.99	3

Significance codes: ^***^0.001; ^**^0.01; ^*^ 0.05; ^ns^0.1.

In the wetter period (August 2022), crown extent was negatively related to predicted percent embolism (coef. = −0.58, SE = 0.06) and Huber value (coef. = −105.3, SE = 2.63), and positively related to height (coef. = 20.38, SE = 0.3). Foliage showed a negative relationship with P_50_ (coef. = −2.78, SE = 0.07) and Huber value (coef. = −289.6, SE = 1.8), and a positive relationship with height (coef. = 31.49, SE = 0.24). The amount of dead leaves was positively associated with osmotic potential at full turgor (coef. = 6.60, SE = 0.43) and the Huber value (coef. = 233.8, SE = 5.6), and negatively associated with height (coef. = −31.5, SE = 0.76). Leaf discolouration was negatively associated with P_50_ (coef. = −1.92, SE = 0.03) and height (coef. = −786, SE = 0.05), but positively associated with predicted percent embolism (coef. = 0.67, SE = 0.00).

## Discussion

Here we quantified the drought tolerance traits and functional responses of six woody species from the Albany Subtropical Thicket, with the aim of improving our knowledge of drought physiological strategies within diverse plant communities and expanding our understanding of Subtropical Thicket ecology. We achieved this aim by testing aspects of the embolism avoidance hypothesis, including the prediction that positive HSM are essential for the maintenance of full canopy function (Skelton et al. 2021). We found that the thicket tree species evaluated were surprisingly drought resistant in terms of xylem functional traits, albeit with some important variation between species. We also show that single traits, such as HSM and P_50_, were weak predictors of drought damage observed during the drought, although they were slightly better predictors of damage following recovery of the vegetation. The strongest of the single trait predictors was the predicted amount of embolism experienced during the drought. Surprisingly, predicted amount of embolism was associated with long-term damage and lack of recovery following rehydration, rather than being a strong predictor of any of the damage metrics during the drought. Functional trait combinations could also be linked to the degree of drought impact that species experienced during a protracted dry period, although our small sample sizes constrain the statistical power of the GLM and limit the generalizability of the findings. Despite these limitations, we suggest that the observed trends provide valuable insights into potential trait–drought damage relationships in Albany Subtropical Thicket, serving as a foundation for future studies with larger sample sizes and broader spatial coverage. Taken together, our results provide support for the embolism avoidance hypothesis, but they also highlight the complexity of predicting drought outcomes within a single major growth form (i.e., woody trees) within a community. The variability of traits could well be driven by the high coefficient of variation associated with moisture availability in the Subtropical Thicket, which might enhance the number of viable functional strategies. Finally, we also provide an explanation for the evergreen nature of this closed-canopy vegetation in a semi-arid region and comment on future predictions for the region.

### Hydraulic traits and functional strategies

Our study describes the interspecific range in capacity to withstand xylem embolism in common woody tree species associated with Albany Subtropical Thicket. It demonstrates that woody thicket species possess xylem with the capacity to withstand very negative water potentials (e.g., P_50_ < −4.5 MPa), placing them among some of the most resistant woody angiosperm species in terms of capacity to withstand embolism. Our findings, taken from a phylogenetically diverse sample of species, suggest that possessing resistant xylem is a pre-requisite for woody shrubs and trees occupying the Albany Subtropical Thicket region. Nevertheless, P_50_ varied twofold among our six co-occurring sample species—as has been observed elsewhere in other plant communities (Pockman and Sperry 2000, Jacobsen et al. 2009, Anderegg et al. 2018, Trugman et al. 2020, Skelton et al. 2021)—further underlining the fact that this trait does not solely determine habitat occupancy or drought susceptibility of a species.

When the xylem vulnerability curve and TLP and P_min_ information was integrated, two functional trait strategies emerged within our community. *Polygala*, *Schotia* and *Pappea* were more drought-avoiding, possessing less negative TLP and P_50_ values in comparison with the more drought-tolerating *Euclea*, *Searsia* and *Boscia*, which had more negative TLP and P_50_ values. *Polygala*, *Schotia* and *Pappea* possessed narrower stomatal safety margins in comparison with *Euclea*, *Searsia* and *Boscia*. Three important findings for drought ecophysiology emerge from these results. Firstly, all six study species possessed positive stomatal safety margins, thus supporting a main prediction of the embolism avoidance hypothesis, namely that stomatal closure should occur before water potentials associated with the formation of xylem embolism. The finding that the stomatal safety margin increased with increasing xylem embolism resistance provides further support for the hypothesis that there exists an absolute limit by which stomata must close to avoid rapid death in drought conditions (Martin-StPaul et al. 2017).

Secondly, our snapshot water potential results showed that the xylem water potential of all sample species was more negative than the water potential associated with turgor loss in both sampling periods. The fact that water potential was more negative than TLP even in the wetter period suggests that the sample species likely spend long periods with their stomata closed. Stomatal closure, while maintaining leaf hydration by reducing water loss during dry periods, will also limit leaf short-term productivity (Chaves 1991).

This finding suggests that the evergreen C_3_ component of the plant community may spend substantial portions of time in a leafed, but dormant state with slower turnover of carbohydrate pools (Zang et al. 2014). Most plants in the thicket are evergreen, which is unusual in that this trait is usually associated with angiosperms in aseasonal regions with high rainfall (e.g., tropical rain or cloud forests), Mediterranean climates, temperate forests or nutrient-poor sites in seasonal subtropics (Givnish 2002). We postulate that the evergreen trait is advantageous in this environment because dry periods and the quantity of rainfall are unpredictable. Maintaining leaves through dry periods ensures a rapid restart to photosynthetic activity after rains (in contrast winter deciduous or drought-deciduous plants may need to first regrow their leaves). Thus, evergreen plants are in a better position to make use of sporadic and low rainfall events that can occur at any time of the year, whereas this window of opportunity is much narrower for deciduous plants.

Thirdly, our finding that all but one of our six study species (*Polygala*) avoided embolism in the wetter period is in accordance with the central claim of the embolism avoidance hypothesis (i.e., that in typical years, woody tree and shrub species—especially those with long-lived, evergreen leaves—might be expected to exhibit positive HSM consistent with the year-round maintenance of full canopy function) (Skelton et al. 2021). Nevertheless, the scenario was different during the dry sampling year: two species (*Polygala* and *Pappea*) had water potentials that were more negative than or converged on P_50_, while the water potential of *Schotia* converged on P_12_. Surpassing the P_50_ threshold has been associated with plant mortality in angiosperms (Urli et al. 2013), leading to the prediction that *Polygala* and *Pappea* will have experienced severe dysfunction in the leaf xylem during the dry year, while *Schotia* will also have been negatively impacted, albeit less severely. *Searsia*, *Euclea* and *Boscia* had large HSMs, avoiding embolism within leaves, and were therefore predicted to fare comparably better than *Schotia, Polygala* and *Pappea*. Large HSMs may imply different drought response strategies, including high xylem resistance, but also deep roots, early stomatal closure or drought deciduousness to limit P_min_. The implications of these strategies might not be equivalent.

### Drought responses: scaling up from functional traits to drought responses in Albany Subtropical Thicket

All six woody species were negatively impacted by the dry period, which is unsurprising given the protracted length of the drought. For example, all species lost foliage and displayed signs of leaf discolouration in the dry period of 2020. However, the species’ responses varied in important ways, which can be linked to their functional strategies, and will likely influence their responses to future droughts. Variation in drought outcomes across species was not strongly associated with variation in individual functional traits such as HSM or P_50_. However, predicted percent embolism was consistently associated with all four drought damage metrics following recovery of the vegetation, supporting the hypothesis that long-term maintenance of canopy health is associated with avoiding embolism during drought periods.

A failure to recover fully following rainfall was most noticeable in *Pappea* and *Polygala*, consistent with our prediction that both species will have experienced severe leaf xylem dysfunction during the dry period. Such a finding extends previous findings showing that leaf gas exchange recovery following natural drought is rapid unless limited by loss of leaf hydraulic conductance in evergreen woody trees (Skelton et al. 2017b). In *Schotia*, crown extent was low during the drought, and individuals were observed to have dead leaves, suggesting that the leaves were also hydraulically impaired. However, both crown extent and foliage amount recovered following the return of rainfall, matching our prediction that this species would have experienced less severe levels of embolism. We suggest that *Schotia* was able to recover by regrowing foliage following the return of rainfall because the xylem transport network had not yet embolized beyond P_12_. *Searsia* and *Euclea*, the two drought-tolerating species with the most resistant xylem and largest HSMs, had no change in crown extent and recovered well following the return of rainfall.

We explored functional trait combinations that could also be linked to the degree of drought impact that species experienced during the protracted dry period. Although our small sample sizes limit the generalizability of the findings, we provide some evidence that leaf construction traits, such as LMA and modulus of elasticity, and plant architecture traits, such as Huber value and tree height, were also associated with drought damage, albeit less predictably so than predicted percent embolism. Surprisingly, our predictive model results indicate that there might be a canopy-level cost to high Huber values. High Huber values are typically thought to favour hydraulic supply over demand, leading to the prediction that species with higher Huber values should experience less drought damage. Instead, species with higher Huber values had lower percentage foliage per branch. These results suggest that there is a potential risk to high sapwood area, namely that if these species experience embolism they may lose their capacity to hydrate their leaves and fail to recover from the drought period. Our results indicated that this scenario occurred in *Pappea*. Tree height was also unpredictably related to drought outcomes, with the relationship between tree height and damage outcomes varying between sampling periods. During the drought period taller trees experienced greater drought damage at both the canopy and leaf levels, but the reverse was observed during the wetter sampling period. Longer path lengths associated with greater tree height might impose greater hydraulic stress on tree function during dry periods, leading to embolism and damage. However, once trees have recovered from water stress, tree height plays a different, yet to be determined role.

As the level of analysis shifted from the canopy toward the leaf level, we observed a change in the suite of traits best related to drought damage. Factors such as modulus of elasticity were negatively associated with the proportion of dead leaves and the amount of leaf discolouration, suggesting that leaves with more rigid cell walls can persist through drought periods better than less rigid leaves.

These findings reveal a complex interplay between leaf construction, longevity and physical tolerance to negative water potentials. More rigid leaves are likely to be evergreen (e.g., *Euclea*) as opposed to deciduous, but not all evergreen species had rigid cells (e.g., *Pappea* and *Polygala*). Species with less rigid, evergreen leaves also tended to be more vulnerable to embolism and experienced greater damage at the leaf level during the drought. *Searsia*—like *Pappea*— displayed high leaf discolouration (i.e., loss of chlorophyll) during the dry period indicating loss of leaf function. However, unlike *Pappea*, our data suggest that *Searsia* leaf degradation (discolouration) was not caused by embolism, but rather by resorption of chlorophyll associated with drought deciduousness. Thus, leaf discolouration in *Searsia* is likely an active physiological process associated with drought deciduousness, arising not in response to embolism, but rather as a mechanism to avoid embolism. Consistent with this conclusion is the fact that leaf discolouration in *Pappea* remained high following rehydration (because dead leaves remained on the individuals), whereas this metric improved in *Searsia* as individuals shed dead leaves and regrew new leaves. *Searsia* also had the lowest LMA values of all six species, further indicating that this species is drought-deciduous, and sacrifices leaves during dry periods.

## Conclusion

African taxa are acutely underrepresented in global hydraulic trait datasets, and this gap limits our understanding of how drought affects species composition and physiological health in southern African ecosystems. Our study indicates that drought causes long-term physiological damage in Albany Subtropical Thicket and is a strong environmental filter for these plant communities. Our study also showed that single functional traits such as HSMs and xylem vulnerability to embolism were insufficient predictors of damage levels at either the leaf or canopy level. However, predicted percent embolism was significantly associated with long-term drought damage at both the leaf and canopy levels. We also reveal the mixed functional strategies of thicket species, especially with regard to leaf construction, leaf longevity and xylem traits, which enhanced our understanding of the drought responses. In general, we conclude that more drought avoiding species, such as *Polygala*, *Schotia* and *Pappea*, are more likely to be adversely impacted by drought events in future than drought tolerating species, like *Searsia*, *Boscia* and *Euclea*. Notably, species that tolerate drought are characterized by resistant xylem and large stomatal safety margins, and can be either drought deciduous or evergreen. We also conclude that more drought-avoiding woody species are nearing their tolerance limits in Subtropical Thicket, while drought-tolerating species show greater resilience to arid conditions. This conclusion is consistent with the observation that the drought tolerance of species appears to be linked to the extent of their geographic distribution into more arid areas (Figure S1 available as Supplementary data at *Tree Physiology* Online). Nevertheless, drought-avoiding species may also prioritize reseeding or resprouting. Consequently, their full recovery potential might only become evident through longer-term monitoring of population dynamics. We also note that it is unclear whether our conclusion that woody drought avoiding species are most susceptible to drought applies to non-woody drought-avoiders (e.g., succulents). Succulent species are co-dominant in Albany Subtropical Thicket and future studies should expand the assessment to include this important functional type.

Finally, we observed that evergreen species in Albany Subtropical Thicket, despite experiencing negative water potentials below their wilting points, maintain their leaves through extended dry periods. This strategy allows for rapid resumption of photosynthesis following unpredictable rainfall if the plant survives the dry period. However, this strategy also poses risks, particularly when coupled with increased herbivory. We postulated that the drought hardiness of evergreen Thicket vegetation is reliant on maintaining its leaves at low costs—where herbivory both reduces the leaf area and increases the costs to the plant in terms of repair and regrowth of leaves. We suspect that this may place plants into carbon debt (Galiano et al. 2011, Poyatos et al. 2013, but see also Sala et al. 2012), where they cannot produce sufficient photosynthate to maintain their structures. Fencing and artificial water sources may have intensified herbivory pressures in dry phases (Healy et al. 2020), contributing to the degradation of this unique ecosystem. Future research should further investigate the interplay between drought and herbivory to develop sustainable management strategies for these ecosystems.

## Supplementary Material

Supplementary_Material_March_2025_tpaf045

## Data Availability

The data underlying this article are available in Harvard Dataverse at https://doi.org/10.7910/DVN/WOHQ9F.
