# Supplementary material for: Mixed hydraulic responses to drought in six common woody species from a dry evergreen sclerophyll forest in South Africa
Source: Tree Physiol. 2025 Apr 16;45(5):tpaf045. doi: 10.1093/treephys/tpaf045 (PMC12106281; doi:10.1093/treephys/tpaf045)
Supplement: Supplementary_Material_March_2025_tpaf045 [file supplementary_material_march_2025_tpaf045.pdf]

Supplementary Material

Figure S1: The geographic distribution of all six study species (south of S22°) against a background of mean annual rainfall.

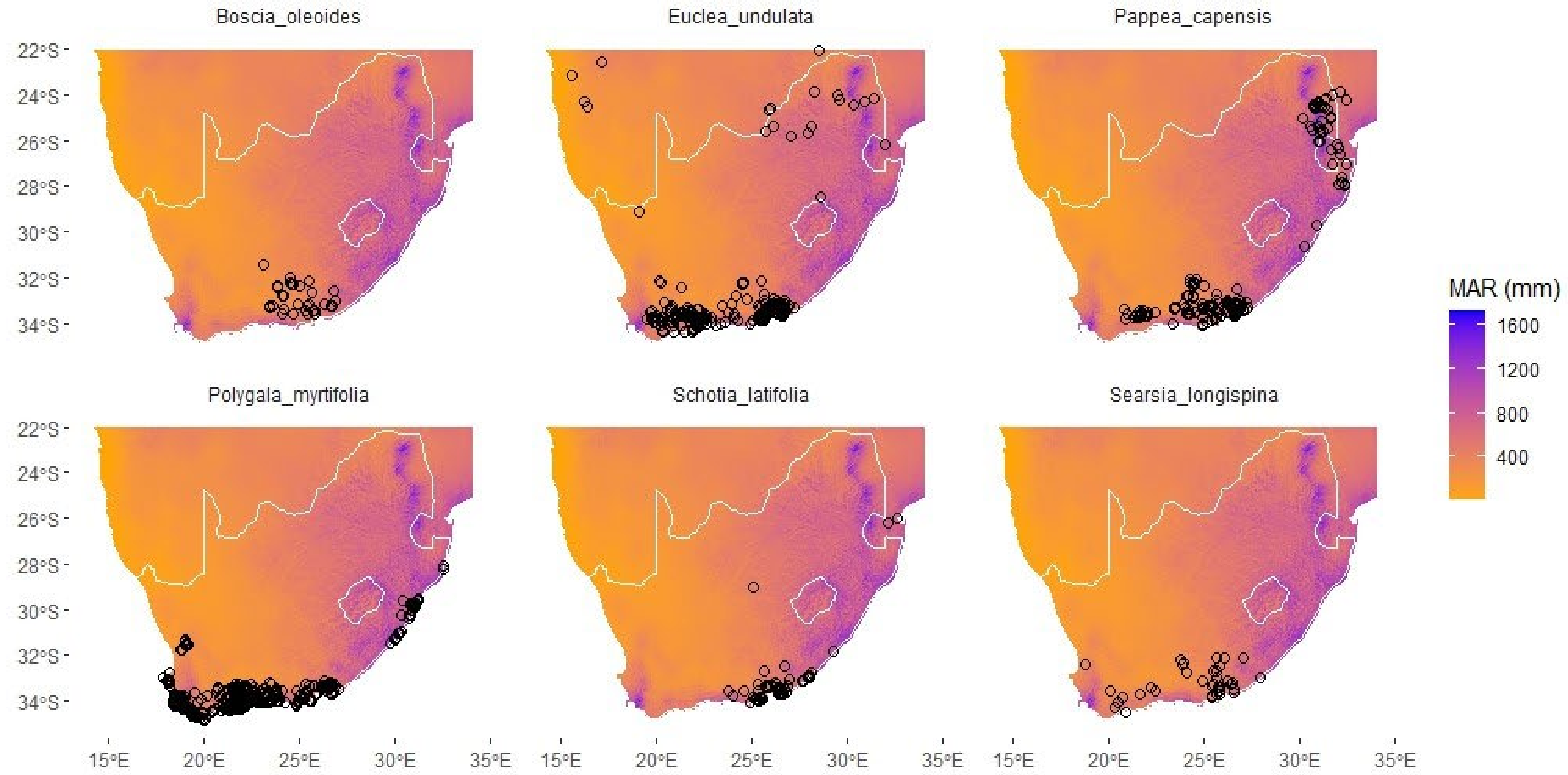

Figure S2: Relationship between leaf mass per area (LMA, g m^{-2}) and leaf P_{50} and TLP for six study species.

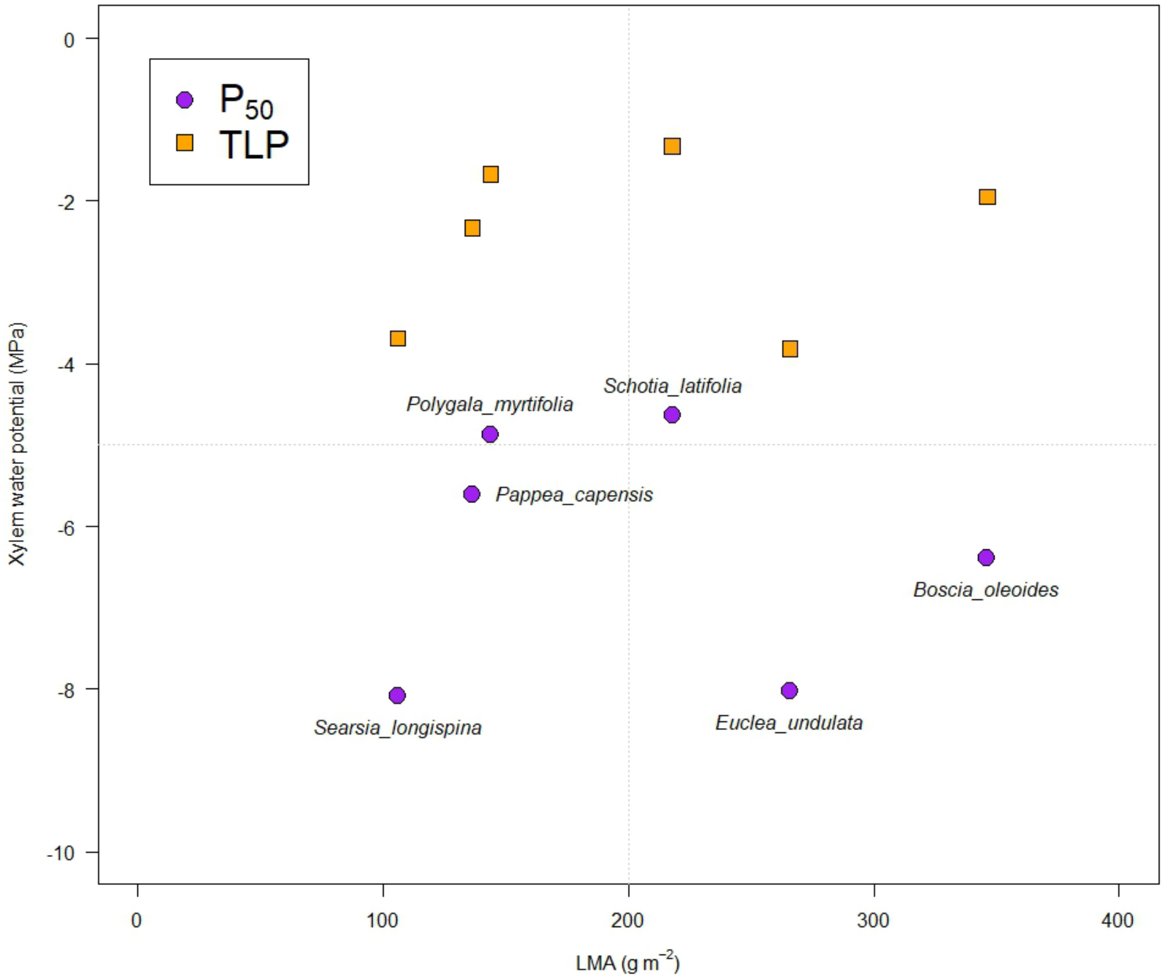

Figure S3: Relationships between hydraulic safety margin from P_{50} and drought damage metrics measured in a drought (October 2020) and a recovery period (August 2022) for six study species.

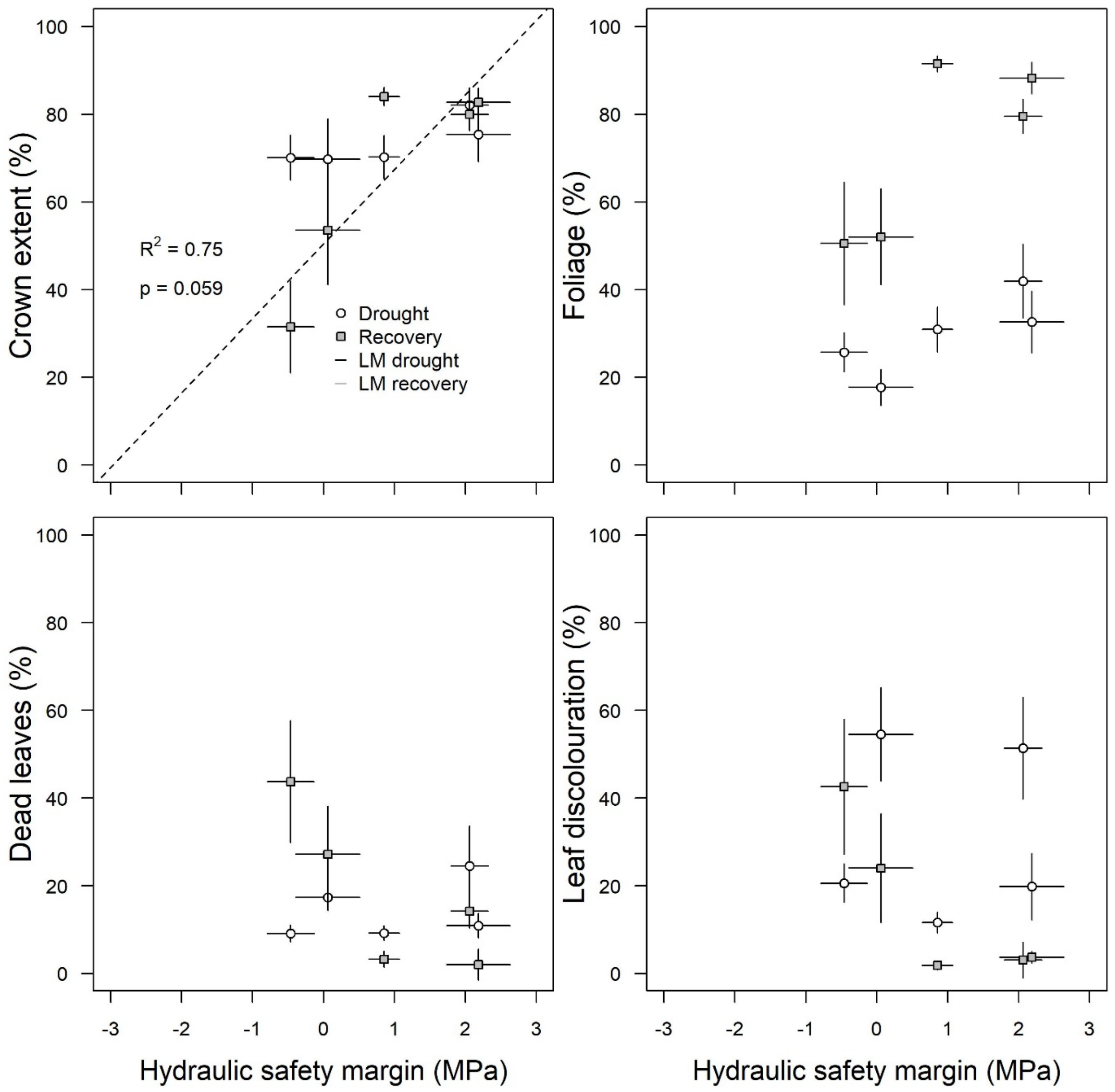

Figure S4: Relationships between hydraulic safety margin from P_e and drought damage metrics measured in a drought (October 2020) and a recovery period (August 2022) for six study species.

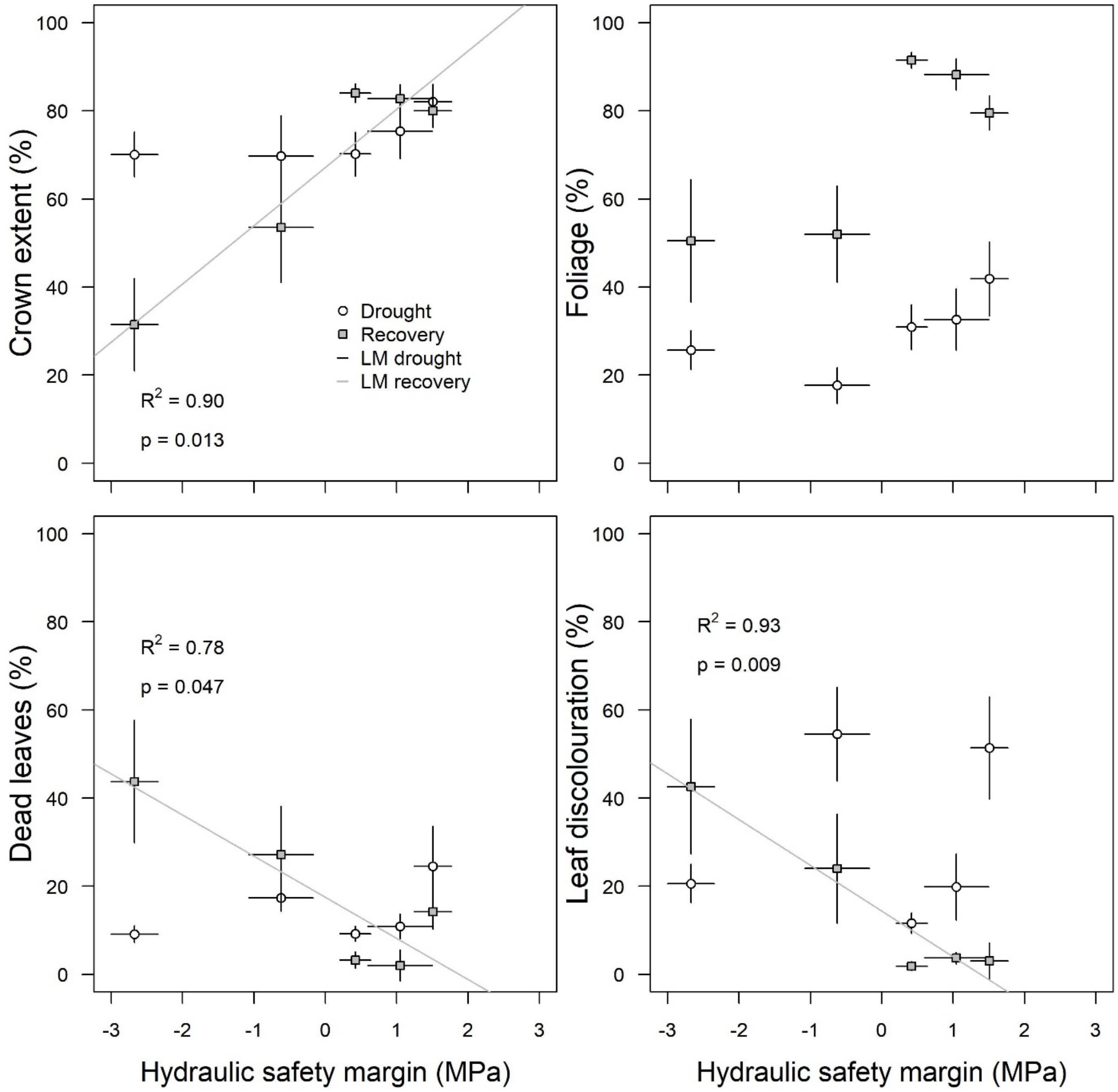

Figure S5: Relationships between xylem vulnerability to embolism (P_{50}) and drought damage metrics measured in a drought (October 2020) and a recovery period (August 2022) for six study species.

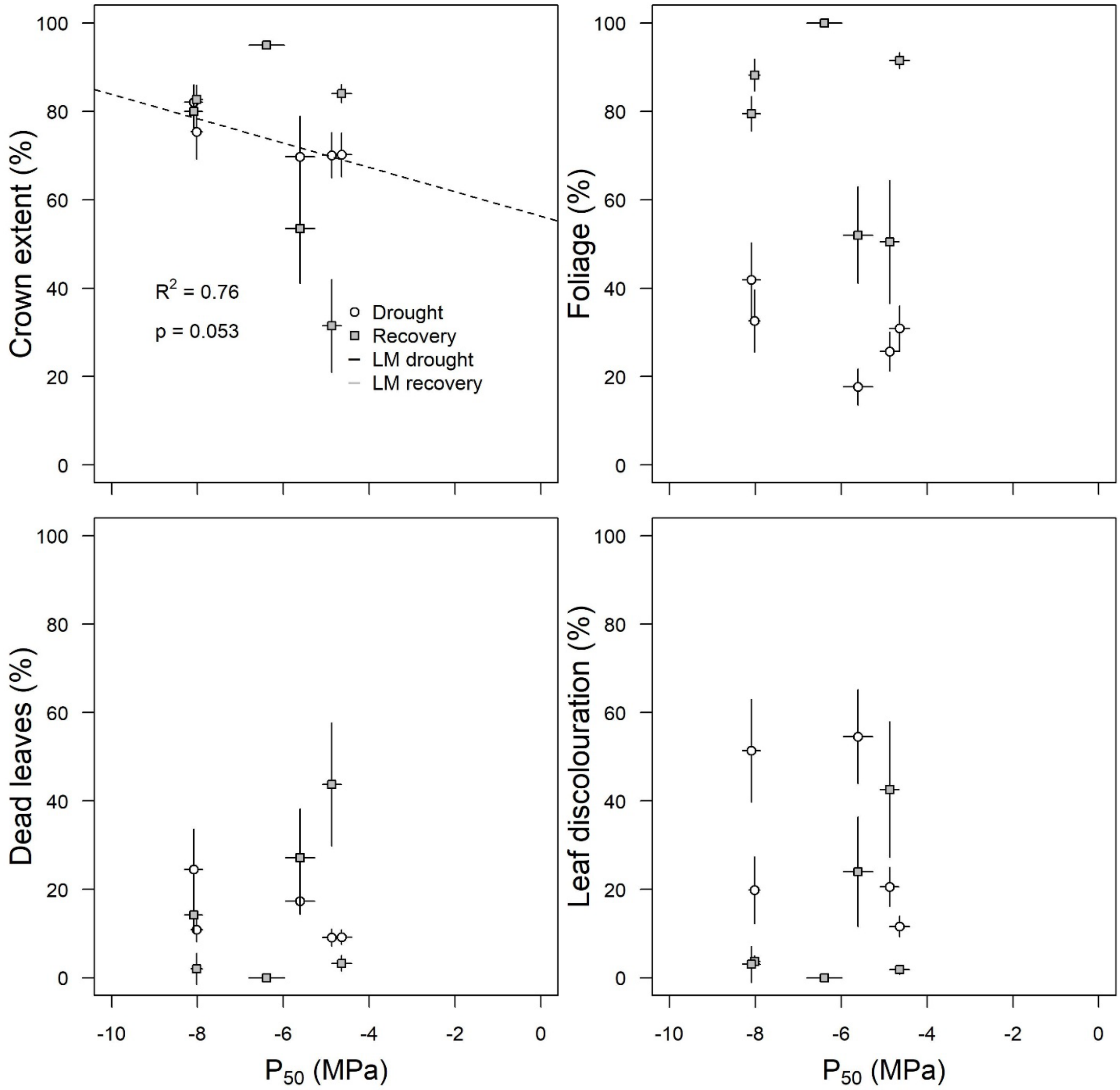

Table S1: Hydraulic safety margins for species. Hydraulic safety margin from the air entry water potential (P_{12}) were quantified in a dry period (August 2020) and in a wetter period (November 2022). Values are mean ± standard error, with sample size in parentheses. Dissimilar letters denote significant differences between species at $p < 0.05$; each trait (per year for HSM) was analysed statistically using one-way ANOVA and post-hoc Tukey HSD.

**Species**	**HSM 2020 (MPa)**	**HSM 2022 (MPa)**	**SSM (MPa)**
Boscia oleoides (Capparaceae)	NA	0.72 ± 0.17 (7) b	2.77 ± 0.20 b
Searsia longispina (Anacardiaceae)	1.12 ± 0.26 (10) c	2.85 ± 0.19 (7) d	3.47 ± 0.11 c
Polygala myrtifolia (Polygalaceae)	−3.03 ± 0.33 (10) a	−1.48 ± 0.15 (7) a	0.64 ± 0.12 a
Pappea capensis (Sapindaceae)	−0.69 ± 0.45 (10) b	0.10 ± 0.21 (7) b	2.54 ± 0.11 b
Euclea undulata (Ebenaceae)	0.92 ± 0.45 (11) c	1.88 ± 0.18 (7) c	2.94 ± 0.04 bc
Schotia latifolia (Fabaceae)	0.21 ± 0.22 (10) bc	0.93 ± 0.30 (8) b	2.71 ± 0.08 b

Supplementary text

Climate data associated with the distribution of each species

Locality data for each species was obtained from GBIF (www.GBIF.org) using the *rgbif* library version 3.7.6 (Chamberlain et al. 2023) in R. The GBIF occurrence download was on 2023-10-26 and is available from here: https://doi.org/10.15468/dl.jj7q4f. The GBIF dataset was filtered to exclude all localities without latitude and longitude values, where the coordinates represented quarter-degree square centroids, and only the indigenous extent of the species distribution (e.g. *Polygala myrtifolia* is endemic to South Africa but is an invasive species on many continents). In addition, *Pappea capensis* localites outside of the Drakensberg coastal hinterland were removed due to a strong genetic break between the southern and northern distribution (likely indicating two separate species, Potts 2012).

Using the filtered GBIF distribution of each species we then used the Chelsa database (ver. 2.1; https://chelsa-climate.org/; Karger et al., 2017, 2021) to identify the climatic conditions associated with the geographic distributions of each point for each species. We extracted the climatic water deficit (CWD, mm) and mean annual precipitation (MAR, mm) associated with each coordinate, from which we calculated the 5th, 50th and 95th percentiles for each species. Climatic water deficit (CWD) is defined as the difference between mean annual precipitation and potential evapotranspiration at each location.

Relationship between P_{50} and environment associated with the geographic distribution of species

The mean P_{50} for each species was not significantly related to the MAP associated with the geographic distribution of the species ($r^2 = 0.36$, $p = 0.21$; Figure 4). The mean P_{50} for each species was also not significantly related to the CWD associated with the geographic distribution of each species ($r^2 = 0.25$, $p = 0.3$). The mean annual historical CWD at the Kaboega study site fell within the range of mean annual conditions across all sites occupied by each species for their entire geographic distributions.

Additional references cited in Supplementary material:

Chamberlain S, Barve V, Mcglinn D, Oldoni D, Desmet P, Geffert L, Ram K (2023). _rgbif: Interface to the Global Biodiversity Information Facility API_. R package version 3.7.6, <https://CRAN.R-project.org/package=rgbif>

Karger DN, Conrad O, Böhner J, Kawohl T, Kreft H, Soria-Auza RW, Zimmermann NE, Linder HP, Kessler M. 2017. Climatologies at high resolution for the earth's land surface areas. *Scientific Data, 4,* 170122.

Karger DN, Conrad O, Böhner J, Kawohl T, Kreft H, Soria-Auza RW, Zimmermann NE, Linder HP, Kessler M. 2021. Climatologies at high resolution for the earth's land surface areas. : 2.1 KB.
